# The ion channel TRPA1 is a modulator of the cocaine reward circuit in the nucleus accumbens

**DOI:** 10.1038/s41380-024-02623-4

**Published:** 2024-05-31

**Authors:** Young-Jung Kim, Su Jeong Choi, Sa-Ik Hong, Jung-Cheol Park, Youyoung Lee, Shi-Xun Ma, Kwang-Hyun Hur, Young Lee, Kyeong-Man Kim, Hyung Kyu Kim, Hee Young Kim, Seok-Yong Lee, Se-Young Choi, Choon-Gon Jang

**Affiliations:** 1 Department of Pharmacology, School of Pharmacy, Sungkyunkwan University, Suwon, 16419 Republic of Korea; 2 Department of Physiology, Dental Research Institute, Seoul National University School of Dentistry, Seoul, 03080 Republic of Korea; 3 Pharmacology Laboratory, College of Pharmacy, Chonnam National University, Gwangju, 61186 Republic of Korea; 4 Department of Physiology, Yonsei University College of Medicine, Seoul, 03722 Republic of Korea

**Keywords:** Addiction, Neuroscience, Physiology

## Abstract

Drug addiction therapies commonly fail because continued drug use promotes the release of excessive and pleasurable dopamine levels. Because the connection between pleasure and drug use becomes hard-wired in the nucleus accumbens (NAc), which interfaces motivation, effective therapies need to modulate this mesolimbic reward system. Here, we report that mice with knockdown of the cation channel TRPA1 (transient receptor potential ankyrin 1) were resistant to the drug-seeking behavior and reward effects of cocaine compared to their wildtype litter mates. In our study, we demonstrate that TRPA1 inhibition in the NAc reduces cocaine activity and dopamine release, and conversely, that TRPA1 is critical for cocaine-induced synaptic strength in dopamine receptor 1-expressing medium spiny neurons. Taken together, our data support that cocaine-induced reward-related behavior and synaptic release of dopamine in the NAc are controlled by TRPA1 and suggest that TRPA1 has therapeutic potential as a target for drug misuse therapies.

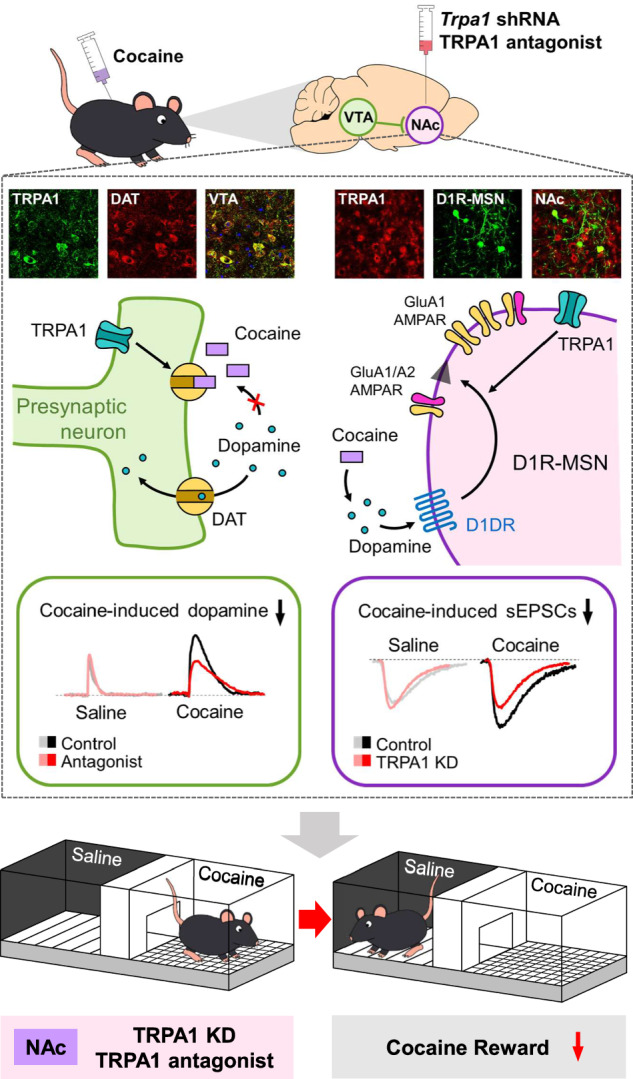

## Introduction

Cocaine is a powerful psychostimulant known to cause severe addiction in users; it enhances drug-seeking behavior to a point where users can lose control of their drug intake. At behavioral and physiological levels, cocaine use leads to compulsory addiction-related behavior and causes long-lasting functional changes in neuronal circuitry [1, 2]. Specifically, cocaine induces an alteration of the reward circuitry in the mesolimbic region [3–5] and increases dopamine concentration in the synaptic cleft by inhibiting dopamine reuptake [6, 7], which enhances neuronal activity in the mesolimbic synapse [8]. The nucleus accumbens (NAc), a neuronal aggregate with an outer shell located in the ventral striatum, is important for reward-related behavior, such as motivation and decision making [9, 10], and is often referred to as the brain’s ‘pleasure center’. Hence, modulation of synaptic transmission in the NAc is believed to be important for the development of addiction-related memory and behavior [11]. At a cellular level, exposure to cocaine enhances the cell surface expression of AMPARs in the NAc [12, 13], and AMPA infusion into the NAc can trigger behavioral responses [14–16]. Furthermore, the medium spiny neurons (MSNs) in the NAc are comprised of two separate populations, demarcated by their expression of different types of dopamine receptors into D1R-expressing and D2R-expressing subtypes (D1R and D2R-MSNs) [17–19]. Considering the crucial role of the NAc, modulators controlling synaptic transmission in the MSNs of the NAc are important candidates for reward/addiction-related functions [13, 20].

To date, dopamine [21], alpha-melanocortin [22], and galanin [23] have been identified as regulators of synaptic transmission in the NAc. Unidentified modulators that alter synaptic transmission in the NAc may also be significant modulators of reward or addiction behavior. Here, we present experimental data supporting that the transient receptor potential ankyrin 1 (TRPA1) is another modulator of neuronal activity of the NAc and of cocaine-related addiction behavior. TRPA1 is a ligand-gated ion channel and a member of the TRP superfamily. It is activated by noxious cold temperatures, extrinsic chemicals such as cinnamaldehyde and menthol, and endogenous agonists such as oxidized lipids like 4-hydroxy-2-nonenal (4-HNE) [24–26] and H_2_S [27]. In both the peripheral and central nervous systems, TRPA1 controls intracellular Ca^2+^ concentrations and membrane depolarization [28–30]. Moreover, emerging evidence indicates that TRPA1 modulates higher brain functions, mood-related conditions, and behaviors such as depression, anxiety, emotion, cognition, and social behavior [31, 32]. Moreover, recent evidence indicates the possible role of TRP family proteins (i.e., TRPV1 and TRPC) in the link between cocaine administration and cocaine-mediated changes in neuronal activity in the NAc [33, 34]. However, it is unknown if TRPA1 affects reward circuits, including those in the NAc. In this study, we showed that both knockdown of TRPA1 expression in the NAc and treatment with a TRPA1 antagonist reduced drug-seeking behavior and reward effects in mice that had received cocaine. Mechanistically, we show that pharmacological inhibition of TRPA1 decreases cocaine-induced increase in DA concentration by rescuing the synaptic dopamine reuptake. Overall, our findings shed new light on not only the mechanisms linking TRPA1 and cocaine-dependent reward behavior, but also on TRPA1’s role as a critical regulator of addiction-related behavior.

## Results

### TRPA1 KD and inhibition of TRPA1 in the NAc regulate cocaine-induced reward

**Fig. 1:**
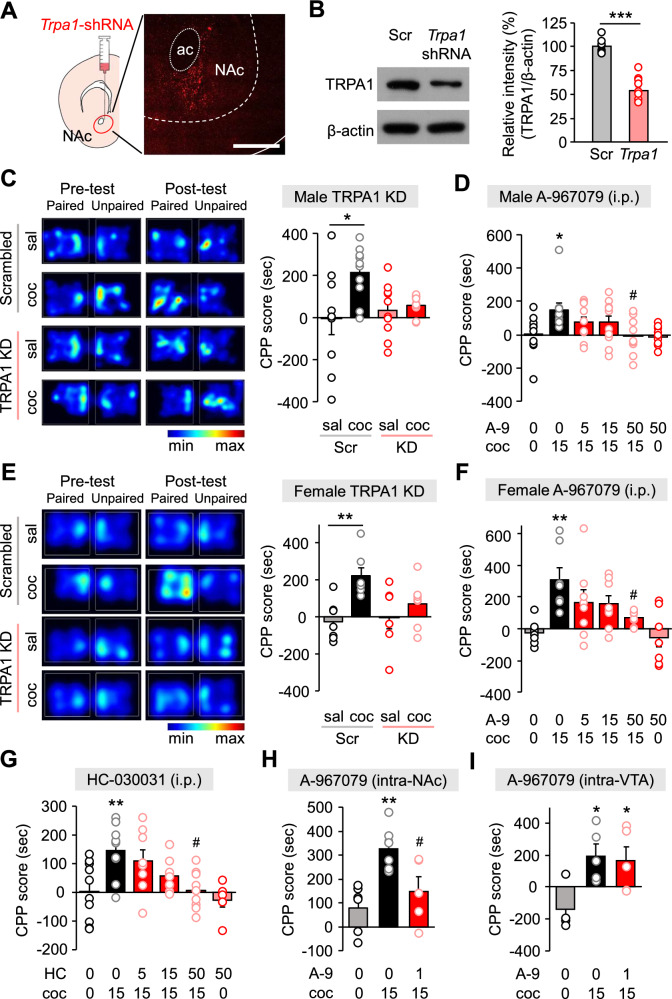
Effects of TRPA1 KD and blockade on cocaine-induced conditioned place preference. **A** Representative confocal image of a coronal of the NAc obtained from *Trpa1*-shRNA-injected mice. Scale bar: 500 μm. ac, anterior commissure. **B** Validation of TRPA1 KD in the NAc of *Trpa1*-shRNA-injected mice by Western blotting (n = 10). **C** Representative heat maps of time spent in each CPP chamber during pre-test and post-test periods of the TRPA1 KD cocaine CPP tests in male mice. CPP score of each group in the TRPA1 KD cocaine CPP tests in male mice (n = 10). **D** CPP score of each group in the TRPA1 antagonist (A-967079) cocaine CPP tests in male mice [n = 10 (control), 10 (cocaine), 10 (A-967079 5 mg/kg, cocaine), 10 (A-967079 15 mg/kg, cocaine), 11 (A-967079 50 mg/kg, cocaine), and 10 (A-967079 50 mg/kg)]. **E** Representative heat maps of time spent in each CPP chamber during pre-test and post-test periods of the TRPA1 KD cocaine CPP tests in female mice. CPP score of each group in the TRPA1 KD cocaine CPP tests in female mice (n = 7). **F** CPP score of each group in the A-967079 cocaine CPP tests in female mice [n = 7 (control), 7 (cocaine), 8 (A-967079 5 mg/kg, cocaine), 8 (A-967079 15 mg/kg, cocaine), 8 (A-967079 50 mg/kg, cocaine), and 8 (A-967079 50 mg/kg)]. **G** CPP score of each group in the TRPA1 antagonist (HC-030031) cocaine CPP tests in male mice [n = 10 (control), 9 (cocaine), 8 (HC-030031 5 mg/kg, cocaine), 10 (HC-030031 15 mg/kg, cocaine), 9 (HC-030031 50 mg/kg, cocaine), and 6 (HC-030031 50 mg/kg)]. **H** CPP score of each group in the intra-NAc injection of A-967079 during cocaine CPP tests in male mice [n = 6 (control), 6 (cocaine), and 5 (A-967079, cocaine)]. **I** CPP score of each group in the intra-VTA injection of A-967079 during cocaine CPP tests in male mice [n = 4 (control), 5 (cocaine), and 5 (A-967079, cocaine)]. Data are presented as mean ± standard error of the mean. *P < 0.05, **P < 0.01, and ***P < 0.001 versus control; ^#^*P* < 0.05 versus cocaine; sal, saline; coc, cocaine; Scrambled or Scr, scrambled control; TRPA1 KD or KD, TRPA1 knockdown; HC, HC-030031; A-9, A-967079.

Repeated drug exposure causes adaptations in the neural reward pathway that reinforce compulsive drug-seeking behavior [3] through mechanisms that synergize with multiple addictive substances [35]. Earlier studies show that repeated cocaine administration alters synaptic transmission in the NAc [36], and that drug-induced reward behavior can be assessed in a memory model linking perception of drug-related pleasure with context-dependent environmental cues [37]. Here, we sought to determine whether altered TRPA1 expression in the NAc affects cocaine reward-related behavior using the conditioned place preference (CPP) paradigm. In this model, mice are administered cocaine in one of two chambers, removed from the set-up, and then reintroduced to assess their preference in chamber. To this end, we stereotactically injected lentivirus expressing *Trpa1*-shRNA tagged with red fluorescence protein (RFP) into the NAc of mice and confirmed that TRPA1 expression was suppressed (Fig. 1A, B). One week following shRNA virus injection, compared to the saline-conditioned group, male mice expressing scrambled shRNA preferred to spend time in the cocaine-paired chamber. In contrast, this was not the case for mice injected with TRPA1 KD shRNA, for which there was no place preference (Fig. 1C). Next, we asked whether pharmacological inhibition of TRPA1 influences cocaine reward. We conducted a cocaine CPP experiment in which male mice were pre-treated with TRPA1 selective antagonist (A-967079) at varying dosages 30 min before cocaine delivery. We found that pre-treatment with A-967079 (50 mg/kg) substantially inhibited the cocaine-induced CPP in male mice (Fig. 1D). We observed that female mice expressing scrambled shRNA show cocaine-induced CPP, but female mice injected with TRPA1 KD shRNA into the NAc did not show place preference (Fig. 1E). We also observed that pre-treatment with A-967079 (50 mg/kg) reduced the cocaine-induced CPP in female mice (Fig. 1F). These results reveal that there is no apparent gender dependence in the contribution of TRPA1 to cocaine reward regulation. We found that pre-treatment with another TRPA1 selective antagonist, HC-030031 (50 mg/kg), substantially inhibited the cocaine-induced CPP (Fig. 1G). In addition, we validated the direct relationship between TRPA1 antagonism in the NAc and regulation of cocaine-induced reward behavior. To this end, we inserted a guide cannula bilaterally in the NAc and delivered A-967079 or vehicle into the NAc 30 min before cocaine injection. Relative to animals pre-treated with vehicle, A-967079 (1 µg/site) injection inhibited cocaine-induced CPP (Fig. 1H). However, TRPA1 antagonism in the VTA generated by A-967079 (1 µg/site) injection into the VTA had no effect on cocaine-induced CPP (Fig. 1I). Among the CPP tests involving the pharmacological inhibition of TRPA1, in groups conditioned with saline, pre-treatment with A-967079 or HC-030031 did not alter CPP score, indicating no addiction-related potential of the TRPA1 antagonists (Fig. 1D, F, G). These findings imply that TRPA1 is mechanistically required for execution of reward-related behavior induced by cocaine and mediated via the NAc.

### TRPA1 in the NAc core affects cocaine-mediated synaptic transmission

**Fig. 2:**
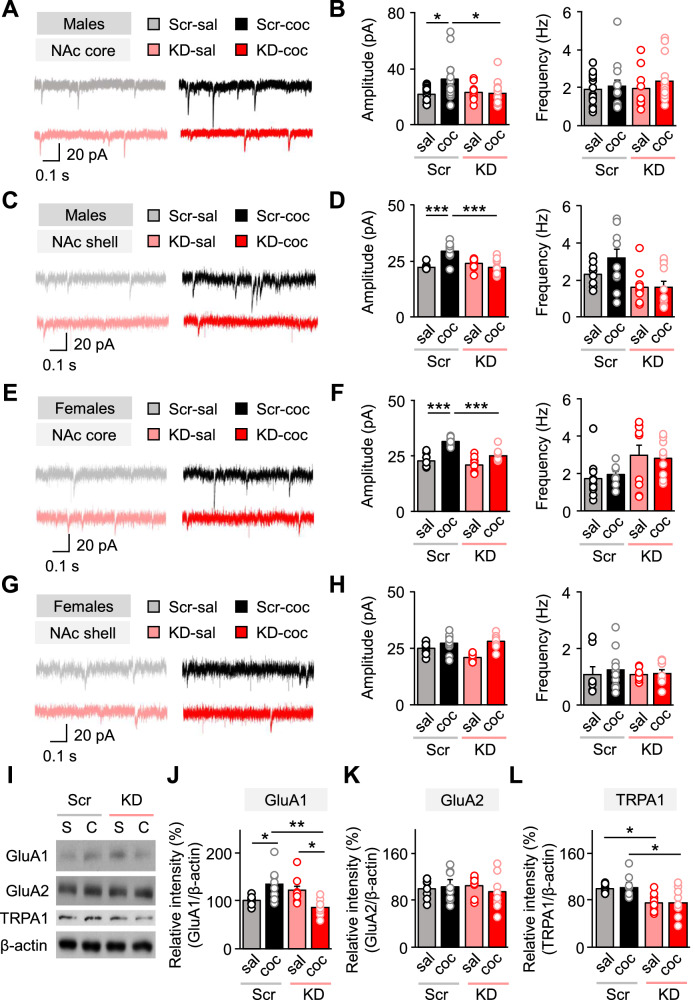
TRPA1 KD disrupted cocaine-effected synaptic transmission in the NAc. **A** Representative raw traces of spontaneous excitatory post-synaptic current (sEPSC) recordings from the NAc core of male mice. **B** Left, sEPSC amplitude in NAc core MSNs of saline- and cocaine-injected scrambled control and TRPA1 KD groups [n = 16 (scrambled, saline), 13 (scrambled, cocaine), 9 (KD, saline), and 15 (KD, cocaine)]. Right, sEPSC frequency in NAc core MSNs of saline- and cocaine-injected scrambled control and TRPA1 KD groups [n = 16 (scrambled, saline), 13 (scrambled, cocaine), 9 (KD, saline), and 15 (KD, cocaine)]. **C** Representative raw traces of spontaneous excitatory post-synaptic current (sEPSC) recordings from the NAc shell of male mice. **D** Left, sEPSC amplitude in NAc shell MSNs of saline- and cocaine-injected scrambled control and TRPA1 KD groups [n = 8 (scrambled, saline), 10 (scrambled, cocaine), 11 (KD, saline), and 11 (KD, cocaine)]. Right, sEPSC frequency in NAc shell MSNs of saline- and cocaine-injected scrambled control and TRPA1 KD groups [n = 8 (scrambled, saline), 10 (scrambled, cocaine), 11 (KD, saline), and 11 (KD, cocaine)]. **E** Representative raw traces of spontaneous excitatory post-synaptic current (sEPSC) recordings from the NAc core of female mice. **F** Left, sEPSC amplitude in NAc core MSNs of saline- and cocaine-injected scrambled control and TRPA1 KD groups [n = 10 (scrambled, saline), 9 (scrambled, cocaine), 9 (KD, saline), and 9 (KD, cocaine)]. Right, sEPSC frequency in NAc core MSNs of saline- and cocaine-injected scrambled control and TRPA1 KD groups [n = 10 (scrambled, saline), 9 (scrambled, cocaine), 9 (KD, saline), and 9 (KD, cocaine)]. **G** Representative raw traces of spontaneous excitatory post-synaptic current (sEPSC) recordings from the NAc shell of female mice. **H** Left, sEPSC amplitude in NAc shell MSNs of saline- and cocaine-injected scrambled control and TRPA1 KD groups [n = 8 (scrambled, saline), 10 (scrambled, cocaine), 10 (KD, saline), and 8 (KD, cocaine)]. Right, sEPSC frequency in NAc shell MSNs of saline- and cocaine-injected scrambled control and TRPA1 KD groups [n = 8 (scrambled, saline), 10 (scrambled, cocaine), 10 (KD, saline), and 8 (KD, cocaine)]. **I** Representative images of Western blot from each protein assayed. **J** Relative GluA1 protein expression in the NAc of the scrambled control and TRPA1 KD groups following cocaine CPP tests in male mice (n = 8). **K** Relative GluA2 protein expression in the NAc of the scrambled control and TRPA1 KD groups following cocaine CPP tests in male mice (n = 8). **L** Relative TRPA1 protein expression in the NAc of the scrambled control and TRPA1 KD groups following cocaine CPP tests in male mice [n = 8 (scrambled) and 9 (KD)]. Data are presented as mean ± standard error of the mean. *P < 0.05, **P < 0.01 and ***P < 0.001; S or sal Saline, C or coc cocaine, Scr Scrambled control, KD TRPA1 knockdown.

To understand how TRPA1 mediates addiction-related behavior, we investigated the synaptic transmission caused by TRPA1 knockdown in the NAc core and shell. To this end, we injected both male and female wild-type mice bilaterally, expressing either scrambled shRNA or *Trpa1*-shRNA. To determine the impact of TRPA1 knockdown on synaptic transmission in the NAc core and shell, we recorded spontaneous AMPAR-mediated excitatory post-synaptic currents (sEPSC) in MSNs from the NAc core and shell of both male and female mice (Fig. 2A–H). As other studies have shown [36, 38], repeated cocaine administration significantly increased the sEPSC amplitude of MSNs in the NAc core of both male and female mice (Fig. 2A, E). However, the sEPSC amplitude of MSNs in the NAc shell only increased in male mice (Fig. 2C) but not in female mice (Fig. 2G). In contrast, cocaine had no effect on sEPSC amplitude in *Trpa1*-shRNA-infected MSNs (Fig. 2B, D, F) and no effect on sEPSC frequency in the NAc core and shell of male mice (Fig. 2B, D) or as the NAc core of female mice (Fig. 2F, H).

In Western blot analysis, we compared TRPA1 protein expression in the NAc of scrambled control and TRPA1 KD male mice after a cocaine CPP test. Our data confirmed that *Trpa1*-shRNA successfully downregulated TRPA1 expression in the NAc in the scrambled control group, whereas cocaine had no effect on TRPA1 expression (Fig. 2I, L). Previous studies reported that cocaine induces changes in the subunit composition of the AMPA glutamate receptor [39–41]. Here, in mice treated with cocaine, we detected an increase in GluA1 protein levels relative to the saline-treated group in mice injected with scrambled control, but this effect was absent in TRPA1 KD mice (Fig. 2I, J). We did not find any changes in GluA2 expression across experimental cohorts (Fig. 2I, K). Collectively, our findings highlight TRPA1 as a critical mediator of AMPAR and intracellular signaling in the NAc in the context of cocaine reward-related behavior.

### TRPA1 KD and TRPA1 antagonist attenuate cocaine-mediated behavioral sensitization

We asked whether TRPA1 mediates the behavioral sensitization due to repeated administration of cocaine by measuring locomotor activity. Behavioral sensitization is a state of neuronal adaptation to drug during the transition to incentivize motivation and distinguish from addiction-related behavior [3]. One week after stereotaxic surgery, mice injected with control shRNA or *Trpa1*-shRNA exhibited no differences in locomotor following saline administration. At day 7, based on analysis of 5-min intervals, we found that locomotor activity after repeated cocaine administration was reduced in TRPA1 KD mice relative to control mice (Fig. S1A). Next, we established that chronic (10 days) intraperitoneal injection of TRPA1 selective antagonist HC-030031 had no impact on locomotor activity or object recognition in the open field test or test for recognition of novel items (Fig. S1B, C). Then, we measured locomotor activity following a single injection of cocaine or repeated injections over 5 days in the presence or absence of HC-030031 (50 mg/kg), administered 30 min prior to injection of cocaine. In mice treated with HC-030031, acute cocaine-associated locomotor activity was substantially inhibited, while locomotor activity following saline injection was not changed relative to the control (Fig. S1D). Similarly, in the mice that received repeated injections, cocaine-induced behavioral sensitization was lower in mice treated with HC-030031 relative to the vehicle pre-treated group (Fig. S1E). Analysis of 5-min intervals on day 5 revealed significant differences between groups in locomotor activity after cocaine injection. HC-030031 pre-treatment had no effect on locomotor activity until cocaine was administered 30 min later (Fig. S1E). These results indicate that TRPA1 affects sensitization separately from addiction-related behaviors such as CPP and self-administration.

### Pharmacological blockade of TRPA1 reduces cocaine reinforcement

**Fig. 3:**
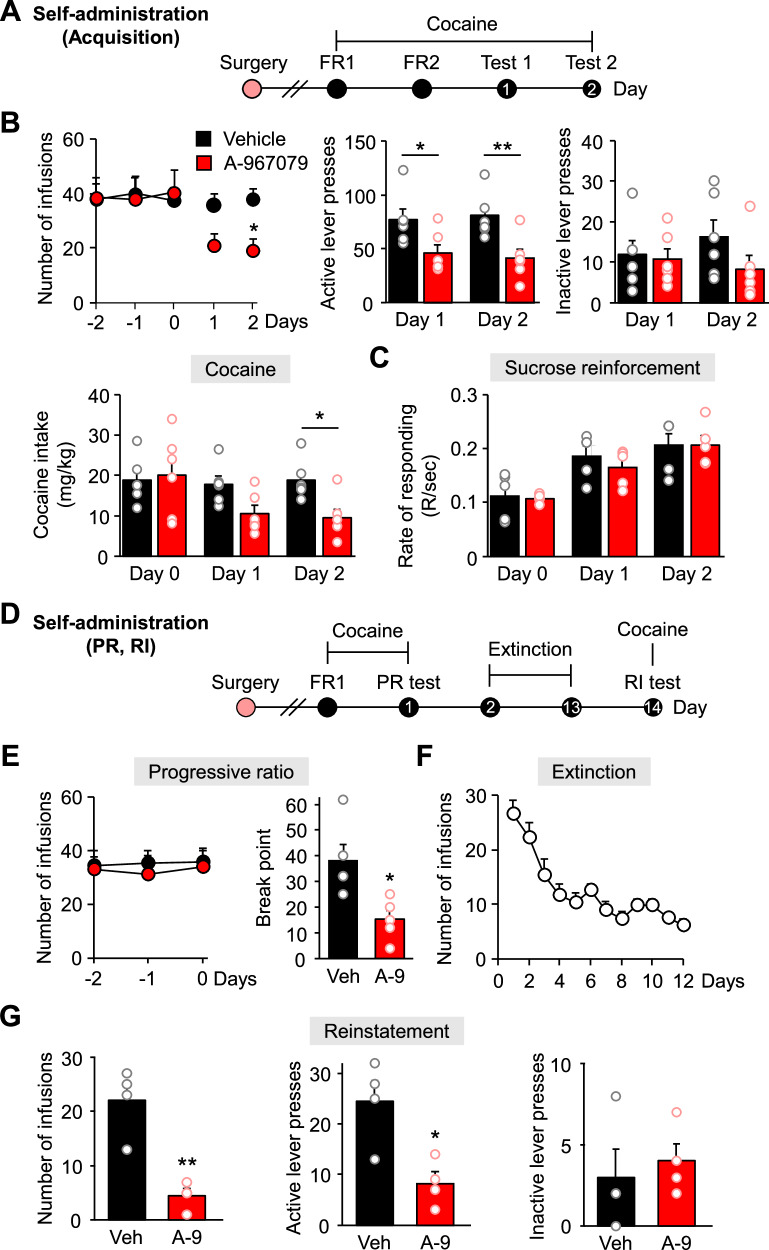
Blockade of TRPA1 reduced cocaine self-administration. **A** Timeline of TRPA1 antagonist cocaine self-administration acquisition test on a fixed-ratio 2 (FR2) schedule. **B** Mean number of infusions from 3 days before test sessions and 2 days of FR2 test sessions following injection of a TRPA1 antagonist (A-967079) or vehicle in a cocaine self-administration test (n = 6). Active lever presses from 2 days of FR2 tests (n = 6). Inactive lever presses from 2 days of FR2 tests (n = 6). Cocaine intake from 1 day before test sessions and 2 days of tests (n = 6). **C** Rate of response to sucrose pellets from 1 day before the test sessions and 2 days of test sessions following injection of A-967079 or vehicle in a sucrose reinforcement test (n = 5). **D** Timeline of TRPA1 antagonist cocaine self-administration test on a progressive ratio (PR) and reinstatement (RI) schedule. **E** Mean number of infusions from 3 days before PR test sessions (n = 5) and breakpoint during the PR test following injection of A-967079 or vehicle in a cocaine self-administration test (n = 5). **F** Mean number of infusions from 12 days of extinction sessions (n = 8). **G** Numbers of infusions (n = 4), active lever presses (n = 4) and inactive lever presses (n = 4) during cocaine-induced reinstatement following injection of A-967079 or vehicle. Data are presented as mean ± standard error of the mean. *P < 0.05 and **P < 0.01 versus control; Veh vehicle, A-9 A-967079.

Using a self-administration paradigm, we examined the influence of TRPA1 inhibition on the regulation of cocaine reinforcement. First, we trained rats to remember a reward action (pressing an active lever that delivers food) and then implanted a catheter into their jugular vein for intravenous infusion of cocaine. After recovery from surgery, rats were trained to self-administer cocaine. Following training, the numbers of lever presses were evaluated, and A-967079 was delivered 30 min before the experiment (Fig. 3A). Three days prior to the experiment, the consistency and number of infusions across groups were comparable. However, upon A-967079 administration, the number of infusions in the second session decreased in the test group relative to the control group (Fig. 3B), and the total number of active lever presses varied between sessions across groups (Fig. 3B). We did not detect any significant variations in inactive lever presses between groups (Fig. 3B). In the second session, the A-967079-treated group consumed less cocaine (Fig. 3B). However, when we used sucrose as a self-administered reinforcer, we found no change in the rate of response between groups treated with vehicle or A-967079 in a sucrose reinforcement test (Fig. 3C).

Next, we investigated if blocking TRPA1 reduces the desire for cocaine. Similar to the previous test, rats were trained to self-administer cocaine and then participated in a progressive ratio (PR) test that required increasingly aggressive lever presses (Fig. 3D). Three days before the test, we verified that both groups experienced a consistent and comparable number of infusions (Fig. 3E). During the PR test, we analyzed the increase in lever presses to receive cocaine infusion and measured the breakpoint. Our data show that the A-967079 group had lower cocaine motivation relative to the control group (Fig. 3E). When we assessed cocaine-seeking behavior in a reinstatement test after withdrawal, we verified that TRPA1 influences cocaine motivation. After the PR test, rats were subjected to 12 days of extinction sessions that had no consequences; all active lever presses across rats were stable at ≤10 responses per session during the last 3 days of the extinction period (Fig. 3F). Then, rats were randomly divided into 2 groups for the reinstatement test. On the day of the test, we administered A-967079, waited 30 min, and evaluated lever presses after a cocaine priming injection. Our data show that A-967079 reduced the number of infusions (Fig. 3G) and active lever presses (Fig. 3G), but there were no changes in inactive lever pressing across groups (Fig. 3G). These findings suggest that cocaine reinforcement is diminished by blocking TRPA1.

### Modulation of TRPA1 manipulates cocaine-induced dopamine release

**Fig. 4:**
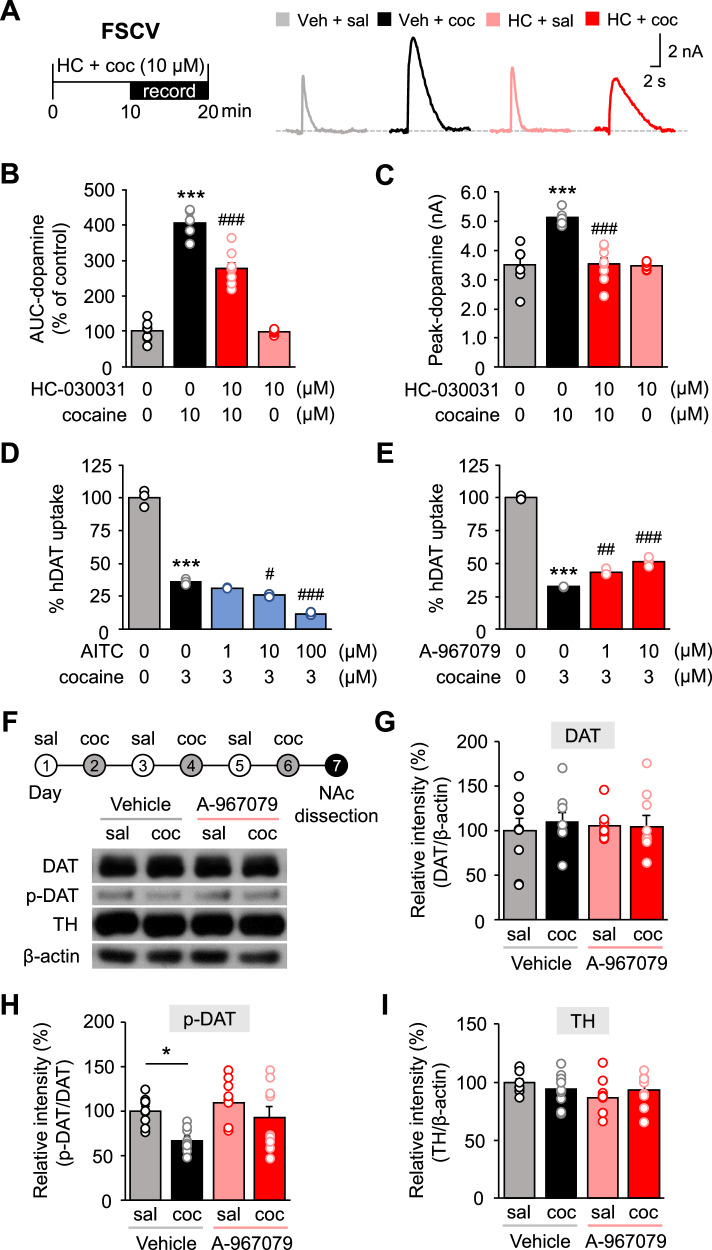
TRPA1 modulation changed dopaminergic activities. **A** Timeline of cocaine and TRPA1 antagonist (HC-030031) application and current recording in FSCV. Representative dopamine voltammetry traces in the NAc core during FSCV. **B** Relative dopamine area under the curve (AUC) in the NAc core of cocaine responses following treatment with HC-030031 or vehicle [n = 7 (control), 5 (cocaine), 8 (HC-030031, cocaine), and 8 (HC-030031)]. **C** Dopamine peak in the NAc core of mice with cocaine responses following treatment with HC-030031 or vehicle [n = 7 (control), 5 (cocaine), 8 (HC-030031, cocaine), and 8 (HC-030031)]. **D** Relative ratio of human dopamine transporter (hDAT) uptake efficacy in hDAT and mouse TRPA1 (mTRPA1) stably transfected HEK293 cells following treatment with cocaine and TRPA1 agonist (AITC) (n = 3). **E** Relative ratio of hDAT uptake efficacy in hDAT and mTRPA1 stably transfected HEK293 cells following treatment with cocaine and TRPA1 antagonist (A-967079) (n = 3). **F** Timeline of cocaine and A-967079 injections, with the NAc dissected 1 day after the last dose. Representative images of Western blot from each protein assayed. **G** Relative DAT protein expression in the NAc of cocaine- and A-967079-injected groups (n = 9). **H** Relative ratio of p-DAT to total DAT protein expression in the NAc of cocaine- and A-967079-injected groups (n = 9). **I** Relative TH protein expression in the NAc of cocaine- and A-967079-injected groups (n = 9). Data are presented as mean ± standard error of the mean. *P < 0.05 and ***P < 0.001 versus control; ^#^*P* < 0.05, ^##^*P* < 0.01, and ^###^*P* < 0.001 versus cocaine; sal saline, coc cocaine, Veh vehicle, HC HC-030031.

Misuse of drugs such as cocaine is highly correlated with the action of the reward pathway comprising dopaminergic neurons in the VTA that project to the NAc. In addition, dopamine release from the nerve endings in the NAc is well known to play a key role in cocaine reward-related behavior [6]. Here, we found that TRPA1 is widely expressed in NeuN-positive neurons in the NAc (Fig. S3A) and also in the VTA (Fig. S3B). In the NAc, TRPA1 was expressed in EYFP-labelled D1R-MSNs (Fig. S3C). In addition, TRPA1 was expressed in dopaminergic neurons in the VAT, which are positively labelled for dopamine transporter (DAT) (Fig. S3D). To investigate whether TRPA1 blockade in the presence of cocaine would alter dopamine levels, we conducted experiments in NAc slices bathed with cocaine and employed fast-scan cyclic voltammetry (FSCV), in which carbon-fiber microelectrodes detect neurotransmitter release in real time at a sub-second time scale. We found that the TRPA1 antagonist HC-030031 (10 μM) reduced both overall dopamine levels released and the magnitude of the dopamine peak induced by cocaine treatment (Fig. 4A–C). Further, in the absence of cocaine, HC-030031 treatment had no direct effect on dopamine levels or peak (Fig. 4A–C), which suggests that blocking TRPA1 did not affect the dopamine level on its own. Cocaine increases dopamine levels by directly binding to the DAT to stop the reuptake of dopamine from the extracellular space [6, 7]. To explore whether TRPA1 modulates cocaine-induced dopamine release, we conducted a dopamine-uptake assay using HEK293 cells transfected with the human dopamine transporter (hDAT) in combination with mouse TRPA1 (mTRPA1). We found that treatment with the TRPA1 selective agonist allyl isothiocyanate (AITC) (10 and 100 μM) in vitro enhanced DAT-dependent inhibitory effects of cocaine in a dose-dependent manner (Fig. 4D). Conversely, TRPA1 antagonist A-967079 (1 and 10 μM) reversed these effects (Fig. 4E). Together, these results support that TRPA1 modulates the affinity with which cocaine binds to the DAT and inhibits dopamine reuptake. Next, to further investigate how TRPA1 regulates the relationship between cocaine and DAT function, we analyzed DAT expression in the NAc of mice following a cocaine exposure after pre-treatment with A-967079 (Fig. 4F). Although we did not observe any changes in DAT expressions by Western blot analysis (Fig. 4G), we detected a reduction in DAT phosphorylation in the cocaine-treated group relative to the saline-treated control, consistent with reduced dopamine reuptake function and plasma membrane trafficking [42, 43]. This effect was reversed in the A-967079 (50 mg/kg) pre-treated group (Fig. 4H). To explore whether the effect of TRPA1 on cocaine-induced dopamine release could be due to alterations in dopamine synthesis, we measured the in NAc levels of tyrosine hydroxylase (TH), the rate-limiting enzyme for dopamine synthesis, and found no change (Fig. 4I). Together, these data suggest a mechanism whereby TRPA1-dependent regulation of dopaminergic activity in the NAc impacts cocaine addiction-related behavior.

### TRPA1 expression does not vary by MSN subtype after exposure to cocaine

**Fig. 5:**
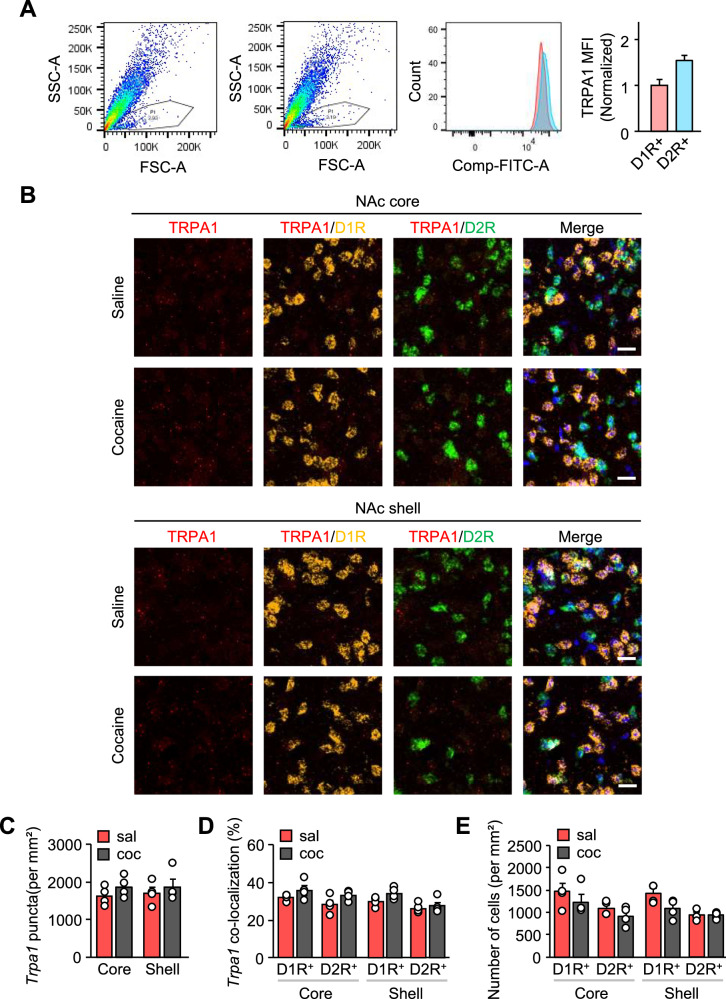
Expression of TRPA1 mRNA in D1R- and D2R-MSNs following cocaine exposure. **A** Representative dot plots and histograms from flow cytometry present the surface expression of TRPA1 of D1R- and D2R- cells in the striatum (n = 3). Bar graphs presented as median defined as the normalized mean fluorescence intensity (MFI) of TRPA1 [n = 3 (WT mice for D1R-MSNs) and 3 (WT mice for D2R-MSNs)]. **B** Representative images of RNAscope showing the distributions of *Trpa1* (red), *Drd1* (yellow), *Drd2* (green), and DAPI (blue) in the NAc. Scale bar: 20 μm. **C** Quantification of the fluorescence of TRPA1 mRNA puncta signals in the NAc subregions of saline- or cocaine-injected mice (n = 4). **D** Quantification of the percentage of D1R- or D2R-MSNs co-localized with TRPA1 signal (n = 4). **E** Quantification of the *Drd1*- or *Drd2*-positive cells (n = 4). Data are presented as mean ± standard error of the mean. sal Saline, coc cocaine.

D1R-MSNs and D2R-MSNs in the NAc express distinct populations of dopamine receptors, and these MSNs have opposing impacts on reward-related behavior. Here, we used flow cytometry to validate the presence of TRPA1 channels in both D1R- and D2R-expressing cells (Fig. 5A). Moreover, to verify whether the expression pattern of TRPA1 is different between D1R-MSNs and D2R-MSNs in the NAc in response to cocaine exposure, we conducted a cell type- and sub region-specific quantitative analysis of TRPA1 mRNA on NAc tissue sections (Fig. 5B). RNAscope fluorescence in situ hybridization analysis revealed that TRPA1 mRNA expression level did not differ between saline- and cocaine-injected mice in either the core or shell region (Fig. 5C). Additionally, the percentage of D1R- or D2R-MSNs co-localized with TRPA1 mRNA was not different in response to cocaine (Fig. 5D). Furthermore, the number of cells expressing D1R or D2R mRNA was not affected by cocaine exposure (Fig. 5E).

### D1R-MSN activity is essential for the impact of TRPA1 KD on cocaine-CPP and synaptic transmission in the NAc core

**Fig. 6:**
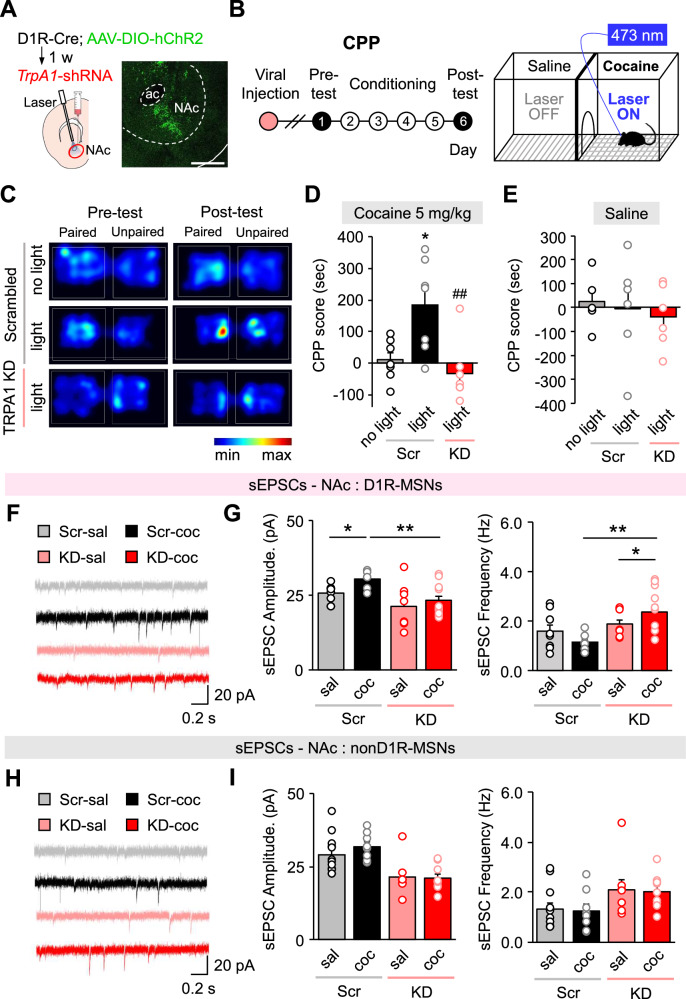
D1R-MSN activity is required for the TRPA1 KD effect on cocaine-CPP. **A** Representative confocal image of a coronal slice of the NAc obtained from AAV-DIO-hChR2-EYFP-injected D1-Cre mice. Scale bar: 500 μm. ac, anterior commissure. **B** Timeline of TRPA1 KD CPP tests with optogenetic activation of D1R MSNs. Schematic representation of the cocaine/blue laser-paired conditioning paradigm. **C** Representative heat map images of time spent in each CPP chamber during pre-test and post-test periods of the TRPA1 KD optogenetic cocaine (5 mg/kg, sub-threshold dose) CPP tests. **D** CPP score of each group in the TRPA1 KD optogenetic cocaine (5 mg/kg, sub-threshold dose) CPP tests (n = 7). **E** CPP score of each group in the TRPA1 KD optogenetic saline CPP tests [n = 5 (control), 6 (scrambled, stimulation), and 6 (TRPA1 KD, stimulation)]. **F** Representative raw traces of spontaneous excitatory post-synaptic current (sEPSC) recordings from D1R-MSNs in the NAc core. **G** Amplitude and frequency of sEPSCs in NAc D1R-MSNs of scrambled control and TRPA1 KD animals by saline and cocaine [n = 8 (D1R-MSNs, scrambled, saline), 6 (D1R-MSNs, scrambled, cocaine), 8 (D1R-MSNs, TRPA1 KD, saline), and 11 (D1R-MSNs, TRPA1 KD, cocaine)]. **H** Representative raw traces of spontaneous excitatory post-synaptic current (sEPSC) recordings from nonD1R-MSNs in the NAc core. **I** Amplitude and frequency of nonD1R-MSNs of scrambled control and TRPA1 KD animals by saline and cocaine [n = 12 (nonD1R-MSNs, scrambled, saline), 8 (nonD1R-MSNs, scrambled, cocaine), 8 (nonD1R-MSNs, TRPA1 KD, saline), and 9 (nonD1R-MSNs, TRPA1 KD, cocaine)]. Data are presented as mean ± standard error of the mean. *P < 0.05 and **P < 0.01 versus control; ^##^*P* < 0.01 versus Scr light, sal saline, coc cocaine, Scr Scrambled control, TRPA1 KD or KD TRPA1 knockdown, w week.

To examine whether TRPA1 KD exerts dopamine receptor-specific effects on cocaine-associated reward behavior, we injected AAV1-Ef1a-DIO-hChR2-EYFP bilaterally into the NAc of D1-Cre mice and selectively activated D1R-MSNs in the cocaine CPP paradigm (Fig. 6A, B). In the absence of cocaine, we observed no difference in CPP score between groups after optical stimulation of D1R-MSNs (Fig. 6E). However, in the presence of cocaine, optical stimulation of D1R-MSNs resulted in a significant increase in CPP score at a subthreshold dose of cocaine (5 mg/kg, Fig. S4A), an effect that was absent in TRPA1 KD mice (Fig. 6C, D).

Glutamatergic input from various regions such as the prefrontal cortex modulates D1R-MSNs and D2R-MSNs in the NAc in distinct ways [44]. Our finding that cocaine-induced AMPAR activity is decreased upon TRPA1-KD raises the question of whether electrophysiological properties of cell-type-specific MSNs contributed to this effect. To test this, we performed patch-clamp electrophysiological recordings in D1-Cre mice (Fig. 6F–I). To label D1R-MSNs in the NAc, we injected AAV1-Ef1a-DIO-EYFP bilaterally into the NAc of D1-Cre mice. Because EYFP is exclusively expressed in D1R-MSNs, this approach allowed us to distinguish between D1R-MSNs (yellow fluorescence) and D2R-MSNs (no fluorescence). Consequently, we observed that the expected cocaine-induced increase in sEPSC amplitude was abolished upon TRPA1 KD in D1R-expressing MSNs (Fig. 6G), while the sEPSC frequency of scrambled control groups was not affected by cocaine (Fig. 6G). The non-D1R-MSN group did not show change in sEPSC amplitude or frequency after cocaine administration (Fig. 6H, I). These results suggest that TRPA1 contributes to the activity of D1R-MSNs in the NAc, which has an important role in mediating the cocaine-associated reward system and behavior.

## Discussion

Modulation of synaptic transmission in the NAc governs cocaine reward-related behavior, but a direct link between TRPA1 and cocaine-associated effects has not been reported. In this study, we explored the role of TRPA1 channels in modulating excitatory synaptic transmissions and cocaine addiction-related behavior as well as dopamine release. We found that both KD of TRPA1 expression in the NAc and TRPA1 antagonist treatment decreased cocaine-seeking and rewarding behavioral responses. Moreover, we detected alterations in cocaine-induced excitatory synaptic activity in NAc MSNs of TRPA1 KD animals. We discovered that TRPA1 KD decreased D1R-MSN-mediated and cocaine-associated reward behavior and reversed excitatory synaptic activity in D1R-MSNs. These results strongly suggest that TRPA1 plays a functional role in the mechanism underlying cocaine reward-related behavior.

TRPA1 contributes to transducing mechanosensitivity and cold sensitivity of sensory neurons [28, 45]. Regulation of TRPA1 channels in relation to pain signaling or neuropathic symptoms has been reported [46]. Although stimulation of TRPA1 is involved in elicited changes in behavioral phenotypes, no link has been demonstrated between TRPA1 and any behavior associated with cocaine reward. Several studies indicate that neither pharmacological blockade nor genetic deletion of TRPA1 alter mechanical pain and thermal (noxious hot and cold) allodynia [47–50]. Moreover, we found that short-term and chronic antagonism of TRPA1 does not affect locomotor behavior, consistent with previous studies that have shown that TRPA1 inhibition and deletion have no effect on motor function [31]. Thus, we conclude the regulatory role of TRPA1 in cocaine-mediated behavioral changes.

The NAc consists of core and shell areas [11]. The NAc shell is related to reward prediction or incentive learning, whereas the NAc core is related to motivation or triggering of drug-seeking behaviors, e.g., initiating movement toward a reward or evaluating substances as rewarding or aversive [51–53]. In addition, the NAc core appears to mediate regulation by conditioned reinforcers, whereas the shell appears to regulate cocaine-induced potentiation [54]. In particular, glutamatergic projections from the prefrontal cortex to the NAc core are implicated in drug-seeking behavior (especially relapse and reinstatement) [55, 56]. For example, if AMPAR transmission is blocked prior to a cocaine challenge due to drug usage, then cocaine-related behavior is also hindered [15]. Numerous research studies have examined how AMPARs and NMDARs, at different levels, alter reward/addiction-related behavior. In previous studies, increased AMPAR transmission induced cocaine-related behavior [12], whereas restoration of baseline transmission was able to abolish the behavior [57]. Here, we confirmed that repeated administration of cocaine increased AMPA-receptor activity in the MSN of the NAc core and shell. However, KD of TRPA1 reduced cocaine-induced AMPAR activity in both NAc core and shell, which explains the mechanism of synaptic transmission associated with cocaine-related behavior upon TRPA1 KD. Notably, these neuroadaptations were reversible since blocking TRPA1 prior to cocaine impact normalized postsynaptic neurotransmission in the NAc.

Foundational studies documented that MSNs in the NAc are involved in addiction-related neuronal mechanisms [36, 58]. D1R- and D2R-MSNs are thought to have distinct actions on reward behavior. The established view is that D1R-MSNs largely encode positive reward-related behavior, whereas D2R-MSNs encode attenuating reward and promote anhedonia and aversive responses [59–62]. We used D1-Cre mice to isolate D1R-MSNs and non D1R-MSNs and to measure the regulatory effect of TRPA1 on cocaine reward responses in specific cell types in the NAc. Here, we asked if D1R-MSN-dependent cocaine addiction-related behavior changed at the synaptic level in the NAc. Upon cocaine exposure, we detected changes in sEPSC amplitude mainly in D1R-MSN, which was rescued in TRPA1 KD mice. Thus, while TRPA1 is expressed in both D1R- and D2R-MSNs, our data support an operational cocaine-associated behavioral effect only in D1R-MSNs. Specifically, cocaine administration leads to the expression of several immediate early genes and the phosphorylation of intracellular signaling proteins only in D1R-MSNs of the NAc [17, 63]. In addition, complementary studies support that the activation of D1R-MSNs increases cocaine reward-context behavior, whereas activation of D2R-MSNs decreases cocaine reward [64–66]. Here, we observed that stimulation of D1R-MSNs in the NAc enhanced cocaine-associated reward behavior, and this effect was blocked by TRPA1 KD in the NAc. As no change in TRPA1 mRNA levels was seen in either D1R- or D2R-MSNs upon cocaine exposure, this reward behavioral phenotype is likely not a consequence of an increase or decrease in TRPA1 expression in the NAc responses to cocaine. Mechanistically, we demonstrate that cocaine intake increases the sEPSC amplitude in D1R-MSNs but does not do so upon TRPA1 KD. These findings indicate TRPA1 as a major regulator of cocaine-mediated synaptic transmission in D1R-MSN of the NAc.

We focused on TRPA1 function in the neurons of the nucleus accumbens. Based on our histology data, NAc MSNs express TRPA1 channels. Although our proposed mechanism for cocaine-induced synaptic transmission involves TRPA1 of MSNs, we cannot exclude the existence and potential function of TRPA1 in non-neuronal cells including astrocytes. Indeed, several studies suggest that TRPA1 is a calcium regulator in hippocampal astrocytes [67], and that astrocytic TRPA1 activity regulates synaptic transmission and plasticity in the hippocampus [68, 69]. In line with these studies are reports that astrocytes in the NAc are linked to ethanol-related reward and motivation by activation of astrocytic D1 receptors [70–72]. The contribution of the TRPA1 channel in astrocytes of the NAc toward synaptic regulation driving reward and addiction remains unknown but merits investigation.

The link between modulation of dopaminergic neurons that project to the NAc and cocaine is an important mechanism for promoting reward behavior [5, 6]. Rewards, such as sucrose or cocaine exposure, rapidly increase dopamine concentration in the NAc [73]. Studies using in vivo photometry have shown that activity of dopaminergic neurons modulates transmission at neuronal terminals in the NAc that contribute to encoding a sucrose reward [74] and also cocaine reward-associated contexts [75]. We observed that inhibition of TRPA1 does not alter sucrose reinforcer behaviors, and other studies reported that TRPA1 blockade is not concomitant with changes in body weight [50], indicating that TRPA1 activity only influences cocaine-dependent responses. It is meaningful to determine whether TRPA1 antagonists are cocaine-like and if TRPA1 antagonists have misuse and/or addiction potential. Our results show that (1) the blockade of TRPA1 itself did not change CPP score or affect dopamine levels like cocaine does, but (2) TRPA1 agonist increased the cocaine’s inhibition on DAT activity, and (3) the TRPA1 antagonist decreased cocaine’s inhibition of DAT activity. These results imply that TRPA1 antagonists are not addictive but could reduce the addiction potential of cocaine. We think that TRPA1 fosters cocaine’s action in presynaptic terminals of dopaminergic neurons in the NAc. Our observations of the cocaine’s action, including those shown in the FSCV and the in vitro assay, imply that TRPA1 is likely changing the action of cocaine on DAT function. We also identified significant expression of TRPA1 in DAT-positive neurons in the VTA. Previous studies have demonstrated that cocaine’s action on DAT has a strong correlation with cytosolic Ca^2+^ concentration or neuronal membrane potential [42, 76–79], which can be modulated by TRPA1 activity. In addition, our data also show that cocaine-induced TRPA1 inhibition reversed DAT phosphorylation. The dopamine transport ability of DAT is tightly regulated by phosphorylation, and cocaine potency is affected by DAT phosphorylation [43, 80, 81]. Several kinases including protein kinase C (PKC) [82, 83] and calcium-calmodulin dependent kinase II (CaMKII) [84] for DAT phosphorylation alter dopamine transport, efflux, and DAT internalization. The activity of intracellular Ca^2+^ influx induced by TRPA1 channel activity could lead to activation of PKC or CaMKII [85, 86]. Increased intercellular Ca^2+^ via depolarization involved in CaMKII activity directly regulates DAT membrane trafficking [87]. As the action of TRPA1 intersects with Ca^2+^ and Ca^2+^-dependent downstream signaling and PKC or CaMKII changes, our results suggest that TRPA1 affects cocaine’s capacity to alter synaptic transmission of dopaminergic terminals or subsequent activation of post synaptic neurons in the NAc to regulate reward-related behavior. Thus, future studies exploring how TRPA1 regulates terminals of DAT-positive neurons extending from the VTA to the NAc likely will provide novel insights into neuronal mechanisms of addiction.

Based on our findings here and several experimental lines of evidence, we conclude that TRPA1 is a novel reward behavior modulator. First, we document abnormalities in cocaine reward-related behavior in TRPA1 KD animals. Second, we show that TRPA1 inhibition reduces cocaine-mediated action on dopaminergic terminals in the NAc. Third, TRPA1 in the NAc regulates the cocaine-induced strength of excitatory synapses, preferentially in D1R-MSNs. Our findings indicate that TRPA1 is a gatekeeper of synaptic transmission in the NAc that governs cocaine-dependent addiction-related responses. As a result of these studies, we hope to contribute to a better understanding of how psychostimulants affect synaptic function and neuronal adaptation.

## Methods

### Animals

Male and female C57BL/6J mice were purchased from Dae Han Biolink, Co., Ltd (Eumseong, Korea) and were used for the TRPA1 KD study, TRPA1 antagonist CPP test, novel object recognition test, immunoblot analysis, immunostaining, flow cytometry, RNAscope and electrophysiology recordings. Male CD-1 mice were purchased from Koatech Co., Ltd. (Pyongtaek, Korea) and were used to determine the effects of TRPA1 antagonist in the locomotor activity and fast scan voltammetry. Male D1-Cre BAC transgenic mice (B6.FVB(Cg)-Tg(Drd1-cre) EY217Gsat/Mmucd) were obtained from Mutant Mouse Resource and Research Center (MMRRC) and were used to investigate the cell-type specific effects of TRPA1 KD. Male Sprague Dawley rats were purchased from Orient Bio Co., Ltd (Seongnam, Korea) and were used to determine the influence of pharmacological manipulation of TRPA1 in the self-administration test. Ten mice were housed per cage (26 × 42 × 18 cm), whereas rats were housed individually in separate cages. All animals were acclimatized in the laboratory animal facility at 24 ± 1 °C and 55 ± 5% humidity under a 12/12 h light/dark cycle, and were used for experiments at 8–10 weeks of age. Animals were allowed access to food and water *ad libitum*, except during food training for rats. All animal care procedures were conducted in accordance with the National Institutes of Health Guide for the Care and Use of Laboratory Animals and approved by the Institutional Animal Care and Use Committees of Sungkyunkwan University.

### Drug preparation

Cocaine hydrochloride (Macfarlan Smith, Edinburgh, UK) was dissolved in saline (0.9% NaCl). HC-030031 (Sigma Aldrich, St. Louis, MO, USA), A-967079 (Enzo Biochem, Inc., New York, NY, USA) and AITC (Sigma Aldrich) were dissolved in vehicle (5% DMSO, 15% tween-80 and 80% saline).

### Stereotaxic surgery

Anesthesia was induced with pentobarbital (50 mg/kg, i.p.) and then mice were placed on a stereotaxic frame (Stoelting Co., Wood Dale, IL, USA). For TRPA1 KD in the NAc, 1 μl of either *Trpa1*-shRNA-lenti-RFP (1 × 10^7^ TU/ml; Origene, Rockville, MD, USA) or scrambled-shRNA-lenti-RFP control virus (1 × 10^7^ TU/ml; TR30033V, Origene) was injected bilaterally into the NAc (AP + 1.5 mm, ML ±1.0 mm and DV −4.5 mm from bregma; 0° angle) of mice at a rate of 0.1 μl/min using a 33-gauge syringe needle. The needle was left in place for an additional 10 min to allow for virus diffusion and then slowly removed. For intra-NAc injection of A-967079, a 26-gauge guide cannulas (P1 Technologies, Roanoke, VA, USA) were implanted bilaterally into the NAc (AP +1.5 mm, ML ±1.0 mm and DV −4.5 mm from bregma; 0° angle). For intra-VTA injection of A-967079, a 26-gauge guide cannulas were implanted bilaterally into the VTA (AP -3.3 mm, ML ±0.5 mm and DV −4.3 mm from bregma; 0° angle). C&B Superbond (Sun Medical, Moriyama, Japan) was applied around the guide cannula to fix on the skull. Two screws were anchored into the skull at the rear of the cannula to help fix the cannula and dental cement (Jet Tooth Shade Powder & Jet Liquid, Lang Dental CO., Wheeling, IL, USA) was applied around the cannulas and screws. For labelling of D1R-MSNs in the NAc, 0.3 μl of AAV1-Ef1a-DIO-EYFP (1 × 10^13^ vg/ml; #27056, Addgene, Watertown, MA, USA) was injected bilaterally into the NAc (AP +1.5 mm, ML ± 1.0 mm and DV −4.5 mm from bregma; 0° angle) of D1-Cre mice at a rate of 0.1 μl/min using a 33-gauge syringe needle. Again, the needle was left in place for an additional 10 min to allow for virus diffusion and then slowly removed. After 1 week of AAV injection, *Trpa1*-shRNA-lenti-RFP was injected bilaterally into the NAc for TRPA1 KD of both D1R- and non-D1R-MSNs. For optical stimulation of D1R-MSNs in the NAc, 1 μl of AAV1-Ef1a-DIO-hChR2-EYFP (7 × 10^12^ vg/ml; #20298, Addgene) was injected bilaterally into the NAc (AP +1.5 mm, ML ±1.0 mm and DV −4.5 mm from bregma; 0° angle) of D1-Cre mice at a rate of 0.1 μl/min using a 33-gauge syringe needle. After 1 week of AAV injection, *Trpa1*-shRNA-lenti-RFP was injected into the NAc and optical cannulas constructed with 200 μm core 0.37 NA optic fiber threaded through 1.25 mm ceramic ferrule (Inper, Hangzhou, China) were implanted bilaterally into the NAc (AP +1.5 mm, ML  mm and DV −4.3 mm from bregma; 10° angle). C&B Superbond was applied around the optic cannula to fix on the skull. Two screws were anchored into the skull at the rear of the optical cannula to help fix the optic cannula and dental cement was applied around the optical cannulas and screws. Mice were allowed to recover and express the virus for at least 1–2 weeks before experiments. During the recovery period, mice were given daily injections of the antibiotic gentamicin sulfate (0.32 mg/kg, i.p.; Shin Poong Pharm Co., Ltd, Seoul, Korea).

### Locomotor activity test

The locomotor activity test was conducted in opaque black plastic boxes (30 × 30 × 30 cm) under dim light (12–13 lux). For the acute cocaine locomotor activity test, mice were first injected with HC-030031 (50 mg/kg, i.p.) or vehicle; 30 min later, mice were injected with saline or cocaine (15 mg/kg, i.p.). Locomotor activity was recorded for 30 min immediately after saline or cocaine injection. For the cocaine behavioral sensitization test, mice were injected with saline during the 2 days of the habituation phase. During the sensitization test phase (days 1–5), mice were injected with HC-030031 (50 mg/kg, i.p.) or vehicle and then, after 30 min, injected with saline or cocaine (15 mg/kg, i.p.). Locomotor activity was recorded for 30 min immediately after saline or cocaine injection. For the cocaine sensitization test with KD of TRPA1 in the NAc, mice were first microinjected with *Trpa1*-shRNA-Lenti or scrambled-shRNA-Lenti into the NAc; the cocaine sensitization test was performed after 1–2 weeks of recovery. After a 2-day habituation phase with saline injections, mice were injected with cocaine (15 mg/kg, i.p.) during the sensitization test phase (days 1–7). As before, locomotor activity was recorded for 30 min immediately after saline or cocaine injection. All behavioral data were recorded using video tracking software (NeuroVision, Busan, Korea).

### CPP test

The CPP apparatus comprised two chambers (15 × 15 × 15 cm) separated by a closable guillotine door. One chamber had white walls with a stainless-steel grid floor, while the other had black walls with a striped floor. Both chambers were illuminated by dim lighting (12–13 lux). The CPP procedure included a pre-test (day 1), a conditioning phase (days 2–7) and a post-test (day 8). For the pre-test and post-test, mice were allowed to access both chambers freely and the amount of time spent on each side was recorded for 20 min. The pre-test data was used to separate mice into groups with relatively equal time spent in each chamber. Afterward, the conditioning phase was conducted daily over 6 consecutive days using a biased procedure. Each mouse was then randomly placed in drug-paired chambers for 45 min with alternate i.p. injections of cocaine or saline. For the cocaine CPP test with KD of TRPA1 in the NAc, mice were microinjected with *Trpa1*-shRNA-Lenti or scrambled-shRNA-Lenti into the NAc and the cocaine CPP test was performed after 1–2 weeks of recovery. Mice were injected with saline or cocaine (15 mg/kg, i.p.) and then placed in the drug-paired chambers. On alternate days, the mice received saline and were placed opposite unpaired chambers. For the cocaine CPP test with systemic injection of TRPA1 antagonists, the mice were first injected with either A-967079 (5, 15 and 50 mg/kg, i.p.) or HC-030031 (5, 15 and 50 mg/kg, i.p.), or vehicle; after 30 min, mice were injected with saline or cocaine (15 mg/kg). On alternate days, the mice received vehicle and saline treatments. For the cocaine CPP test with intra-NAc or intra-VTA injection of TRPA1 antagonists, mice were microinjected with 1 μl of A-967079 (1 μg/site) or vehicle into the NAc or VTA through implanted cannulas at a rate of 0.5 μl/min; 30 min later, mice received a saline or cocaine (15 mg/kg) injection. On alternate days, mice received vehicle into the NAc or VTA and saline. The CPP score was calculated by differences between the time spent in the drug-paired chambers during the pre-test and during the post-test. All behavioral data were recorded using video tracking software (NeuroVision).

### Self-administration test

Self-administration tests were conducted using operant conditioning chambers (28 × 26 × 20 cm), which were located in light- and sound-attenuating cubicles (Med Associates, ST. Albans, VT, USA). Housing light, two response levers (active and inactive levers) and two cue lights above each response lever were placed in the operant chambers. For food training, rats were initially trained to active lever press for 45 mg of food pellets (F0021, Bio-Serv, Flemington, NJ, USA) on a fixed-ratio 1 (FR1) schedule until they obtained 80 food pellets during a 1-h session each day for three consecutive days. For catheter implantation surgery, rats were anaesthetized with pentobarbital (50 mg/kg, i.p.) and the catheter (0.3 mm ID × 0.64 mm OD; Dow Corning, Midland, TX, USA) was inserted into their jugular vein. The distal end of the catheter was connected to the back of the rat and exited the skin through a 22-gauge guide cannula (P1 Technologies) anchored with Mersilene surgical mesh (Ethicon Inc., Somerville, NJ, USA). During the recovery period, rats were intravenously injected daily with 0.2 ml of heparin (20 IU/ml) and the antibiotic gentamicin sulfate (0.32 mg/ml) for 7 days. Following the recovery period, rats were trained for a cocaine self-administration test via 2-h daily cocaine [0.5 mg/kg/infusion, intravenous (i.v.)] self-administration sessions with an FR1 schedule over 5 consecutive days and then with an FR2 schedule over 7 consecutive days. An active lever press resulted in presentation of a drug infusion and then a 20-s time-out period during which lever presses were recorded but had no consequences. Only rats that showed a stable number of infusions were used to test the effects of TRPA1 on cocaine reinforcement. Each chosen rat received A-967079 (15 mg/kg, i.p.) or vehicle 30 min before a test, and 2-h daily FR2 test sessions occurred for 2 days. For the progressive ratio (PR) test and reinstatement test, rats were trained in 2-h daily cocaine (0.5 mg/kg/infusion, i.v.) self-administration sessions under an FR1 schedule for 10 consecutive days following recovery. Only rats that showed a stable number of infusions were used in the PR test. Each chosen rat received A-967079 (15 mg/kg, i.p.) or vehicle 30 min before a test, and a 6-h PR schedule session was performed. In PR schedule, active lever press requirements for drug infusion were progressively increased according to the following series: 1, 2, 4, 6, 9, 12, 15, 20, 25, 32, 40, 50, 62, 77, 95, 118, 145, and 178. The breakpoint was defined as the last active lever press requirement for drug infusion. After the PR test, rats underwent daily 1-h extinction sessions for 12 consecutive days. During the extinction session, lever presses were recorded but no responses were delivered. On the following day, rats underwent a cocaine-primed (10 mg/kg, i.v.) reinstatement test in which lever presses were recorded but no responses were delivered. Each rat received A-967079 (15 mg/kg, i.p.) or vehicle 30 min before the test. For the sucrose reinforcement test, rats were trained to active lever-press for 45 mg of sucrose pellets (F0023, Bio-Serv) on a FR1 schedule until they obtained 80 sucrose pellets during a 1-h session each day for three consecutive days. Following training, rats received A-967079 (15 mg/kg, i.p.) or vehicle 30 min before a test, and 1-h daily sucrose reinforcement test sessions occurred for 2 days. All behavioral data were recorded using Med Associates software (Med Associates).

### In vivo Optogenetic stimulation for CPP test

For cocaine CPP test with optical stimulation of D1R-MSNs in the NAc, mice were injected with subthreshold dose of cocaine (5 mg/kg, i.p.) and the 200 μm patch cords were connected to implanted optical fibers using a mating sleeve and then placed in the paired chambers for conditioning. The patch cords were connected to 40 mW 473 nm blue laser (BL473T3-050FC, Shanghai Laser, Shanghai, China) and stimulation pulses were controlled through a waveform generator (33511B, Agilent, Santa Clara, CA, USA). Optical stimulation consisted of 20 Hz frequency, 5 ms pulse duration, and 2–5 mW of light power. Laser pulses were delivered for 3 min period and 5 min rest and repeated for optical stimulation of D1R-MSNs in NAc. On alternate days, mice received saline and also patch cords were connected and were placed opposite unpaired chambers without optical stimulation. For optogenetic CPP test without cocaine, mice were placed in the paired chambers for conditioning with the connection of patch cords to implanted optical fibers and laser pulses were delivered with same procedure as described previously except saline or cocaine injection. Each mouse was placed in chambers for 45 min and the conditioning phase was performed daily over 4 consecutive days. The CPP score was calculated by differences between the time spent in the drug-paired chambers during the pre-test and during the post-test. All behavioral data were recorded using video tracking software (EthoVision, Noldus, Wageningen, Netherlands).

### Novel object recognition test

HC-030031 was administered at the dose of 10 mg/kg, i.p. once a day for 10 days. Control mice received vehicle. Behavioral test was performed at last 3 day of drug administration period. The novel object recognition test was performed using 9- or 10-week-old male mice. The mice were first placed in an open-field plastic box (22 × 27 × 30 cm) and allowed to freely move for 7 min (habituation). Next day, the mice were placed in the box where two identical objects are placed at the two corners and allowed to explore freely for 5 min (familiarization phase). 24 h later, an object identical to those in the familiarization phase and a novel object were placed in the same location as familiarization phase. Then, mice were reintroduced to the box and allowed to explore freely for 5 min (test phase). Object exploration was scored only when the animal’s snout was directly facing and sniffing the objects. Exploration was not scored if another parts of the animal’s body contacted the objects. All of the objects were cleaned with 70% ethanol between animals to remove odor cues. The observer was blinded to the type of drug administered to the animal. Discrimination ratio was calculated as follows: (exploration time with novel object − exploration time with familiar object)/(total exploration time). During the habituation, distance moved was analyzed and used to determine animal’s locomotor activity.

### Immunostaining

Mice were deeply anesthetized with pentobarbital (50 mg/kg, i.p.) and intracardially perfused with 4% paraformaldehyde in 0.1-M phosphate-buffered saline (PBS) solution. Their brains were then rapidly dissected and fixed for 4 h with ice-cold 4% paraformaldehyde in 0.1-M PBS. The brains were then dehydrated in 30% sucrose in 0.1-M PBS for 2 days at 4 °C, after which they were frozen. Slices of brain were sectioned coronally to 30-μm thicknesses using a cryostat (Leica, Nussloch, Germany). For immunostaining of target proteins, the slices were first washed three times in PBS and then blocked for 1-h in a blocking solution containing 10% bovine serum albumen and 0.3-M glycine in PBS at room temperature. Subsequently, they were incubated with primary antibodies against TRPA1 (rabbit, Novus, Centennial, CO, USA, NB110-40763, 1:100) and NeuN (mouse, Millipore, Burlington, MA, USA, MAB377, 1:100) or DAT (mouse, Abcam, Cambridge, UK, ab128848, 1:100) at 4 °C overnight. The following day, the slices were washed three times in PBS and incubated with the appropriate secondary fluorescent antibodies (Invitrogen, Waltham, MA, USA, 1:500) for 1-h at room temperature. Finally, the slices were washed three times in PBS and mounted onto microscope slides with H-1500 DAPI or H-1400 non-DAPI containing mounting medium (Vector Laboratories, Burlingame, CA, USA). Digital images were acquired using an LSM 700 laser scanning confocal microscope (Carl Zeiss, Heidelberg, Germany).

### Immunoblot analysis

For immunoblotting, brains were rapidly dissected and then slices of the NAc were sectioned coronally at 800-μm thicknesses using a cryostat (Leica). The NAc were isolated with a 17-gauge stainless-steel punch and NAc tissues were homogenized in ice-cold lysis T-PER (Tissue Protein Extraction Reagent; Thermo Fisher Scientific, Rockford, IL, USA) containing phosphatase and protease inhibitor cocktails (Roche Diagnostics, Indianapolis, IN, USA). After centrifugation at 13,000 × *g* for 15 min, the supernatants were collected. Protein concentrations were determined using a Pierce BCA Protein Assay Kit (Thermo Fisher Scientific). Using 10% sodium dodecyl sulfate-polyacrylamide gel electrophoresis, 5–8 μg of protein was separated and then transferred onto a polyvinylidene difluoride membrane (Millipore). Each membrane was blocked in Tris-buffered saline containing 5% non-fat milk and 0.1% Tween 20 for 1-h at room temperature. The membranes were then immunoblotted with primary antibodies against TRPA1 (rabbit, Novus, NB110-40763, 1:1000), DAT (rabbit, Millipore, AB2231, 1:3000), p-DAT (rabbit, Abcam, ab183486, 1:1000), TH (rabbit, Abcam, ab112, 1:5000), GluA1 (rabbit, Abcam, ab31232, 1:1000), GluA2 (rabbit, Abcam, ab20673, 1:1000) and ß-actin (mouse, Santa Cruz, Dallas, TX, USA, SC-47778, 1:2000) overnight at 4 °C. After the membranes were washed, the blots were exposed to horseradish peroxidase-conjugated secondary antibodies (Jackson ImmunoResearch Laboratories Inc., West Grove, PA, USA) for 1-h at room temperature. The antibody binding was visualized using the enhanced chemiluminescence detection method (Dongin LS., Seoul, Korea) and then exposed to photographic film. Protein bands were quantified by densitometric analysis using ImageJ software (NIH, Bethesda, MD, USA).

### Fast scan cyclic voltammetry

Mice were anesthetized with isoflurane and their brains were rapidly removed. Coronal slices (400-μm thicknesses) of the NAc were maintained at room temperature in oxygenated (95% O_2_ and 5% CO_2_) artificial cerebrospinal fluid, which consisted of NaCl (126 mM), NaHCO_3_ (25 mM), d-glucose (11 mM), KCl (2.5 mM), CaCl_2_ (2.4 mM), MgCl_2_ (1.2 mM), NaH_2_PO_4_ (1.2 mM) and L-ascorbic acid (0.4 mM), pH-adjusted to 7.2. A capillary glass-based carbon-fiber electrode was positioned 75-μm below the surface of the slice in the NAc. DA release was evoked every 2 min by a 4-ms one-pulse stimulation (monophasic, 300 μA) from a stimulating electrode placed 100–200 μm from the carbon-fiber electrode. Drugs were applied via their addition to the perfusion solution (2 ml/min). The electrode potential was linearly scanned as a triangular waveform from −0.4 to 1.3 V and back to −0.4 V versus Ag/AgCl using a scan rate of 400 V/s. Cyclic voltammograms were recorded at the carbon-fiber electrode every 100 ms using a custom-modified headstage connected to a Multiclamp 700B amplifier (Molecular Devices, Sunnyvale, CA, USA) and pCLAMP 10 data acquisition software (Molecular Devices).

### Dopamine uptake assay

Human embryonic kidney-293 (HEK-293) cells were cultured in minimal essential medium supplemented with 10% fetal bovine serum, 100-U/ml penicillin and 100-µg/ml streptomycin in a humidified atmosphere of 5% CO_2_. The cells were transfected with a combination hDAT and mTRPA1 in plasmid vector using the calcium phosphate method. A dopamine uptake assay was performed in HEK-293 cells, which stably express hDAT and mTRPA1. After 48 h of transfection, the medium was removed and cells were rinsed with uptake buffer (5-mM Tris base, 7.5-mM HEPES, 120-mM NaCl, 5.4-mM KCl, 1.2-mM CaCl_2_, 1.2-mM MgSO_4_, 1-mM ascorbic acid and 5-mM glucose; pH 7.1). The cells were then incubated for 5 min at 37 °C with 100 μl of uptake buffer containing 20-nM [^3^H]-dopamine. To measure dopamine uptake, the cells were rinsed with 1 ml of ice-cold uptake buffer three times and then solubilized in 0.5 ml of 1% sodium dodecyl sulfate. Subsequently, the radioactivity was measured by liquid scintillation counting. To determine the effects of TRPA1 agonist and antagonists on dopamine uptake, cells were incubated with corresponding drugs for 20 min and then a dopamine uptake assay was performed.

### Enzymatic tissue dissociation

Two or three wild-type mice at 2 months were anesthetized with isoflurane and decapitated rapidly. Brains were removed and sectioned in the coronal plane at a thickness of 300 μm on the Vibratome in 124-mM NaCl, 5-mM KCl, 1.23-mM NaH_2_PO_4_, 2.5-mM CaCl_2_, 1.5-mM MgCl_2_, 26-mM NaHCO_3_ and 10-mM dextrose, and bubbled with 5% CO_2_/95% O_2_. Total striatum was dissected from brain slices and dissociated for 45 minutes at 37 °C using Papain enzyme (Papain Dissociation System, Worthington Biochem) in Earls Balanced Salt Solution (EBSS) with DNase, per manufacturer’s instructions. Triturating brain slices using three glass pipettes of decreasing tip diameter and centrifuging dissociated cells at 900 rpm for 5 minutes at room temperature. To remove excess debris, cell pellets were resuspended in EBSS, DNase, and albumin ovomucoid inhibitor (AOI), and the cell suspension was centrifuged on an AOI discontinuous gradient at 900 rpm and 4 °C (according to the Papain Dissociation System procedure). Cell pellets were resuspended in buffer medium (L15-CO_2_ without phenol, 1× Pen-Strep, 10 mM Hepes, 25 g/ml DNase, 1 mg/ml BSA) and filtered through a 40 µm (BD Falcon, #352350) mesh. Cells were stained with live-dead straining (Ghost Dye Violet 510, detection of AmCyan) and incubated on ice for 30 minutes protected from light.

### Flow cytometry

To evaluate the TRPA1 channel expression, we used flow cytometry in D1R+ and D2R+ cells from the whole striatum of mouse brains. The cells were washed twice with 1% BSA in phosphate-buffered saline (PBS) and blocked with 2% BSA in PBS. After blocking, the cells were washed and resuspended in Alexa Fluor 647 Conjugated-Dopamine D1 Receptor (Bioss, bs-10612R-A647, 1:100) or Alex Fluor 647 Conjugated-Dopamine D2 Receptor (Santa Cruz Biotechnology, sc-5303, 1:100) with Anti-TRPA1 (extracellular)-FITC antibody (alomone labs, AC-037-F, 1:100) and incubated for 30 min at 4 °C in the dark. All phases of washing were performed using 1% BSA in PBS as the washing buffer, followed by centrifugation (8000 rpm, 1 min, 4 °C). Prior to examination, the labelled cells were stored on ice. For cell analysis, a high-performance Flow Cytometer (LSR Fortessa X-20, Beckton Dickinson, USA) and FlowJo (BD, USA) were used.

### Fluorescence in situ hybridization (RNAscope)

Mice were injected with saline or cocaine (15 mg/kg, i.p.) on alternate days for 6 days related to the cocaine CPP test while control animals received saline injection for 6 consecutive days. 24 h after last injection, mice were transcardially perfused with PBS, and their brains were then harvested and snap-frozen on dry ice. Coronal sections (14-μm thickness) containing the NAc were cut using cryostat (Leica) at −20 °C and mounted on Superfrost plus^TM^ microscope glass slide (Thermo Fisher Scientific). Slide samples were stored at −80 °C. Fluorescence in situ hybridization was performed using RNAscope multiplex fluorescent reagent kit v2 [Advanced Cell Diagnostics (ACD), Newark, CA, USA], as manufacturer’s instructions. Briefly, sections were fixed in 4% paraformaldehyde for 1 h at 4 °C and rinsed with PBS. Sections were then dehydrated with 50% (5 min), 70% (5 min), and 100% (5 min, two times) ethanol. After air drying for 5 min at RT, a hydrophobic barrier was drawn around the sections using a hydrophobic pen (Vector Laboratories) and allowed to dry for 5 min. Sections were then incubated with RNAscope hydrogen peroxide for 10 min at RT and washed with distilled water. Sections were then incubated with RNAscope protease III for 30 min at RT and washed with distilled water, before being incubated with the appropriate probes for 2 h at 40 °C in the HybEZ oven. The following probes were purchased from ACD: Mm-*Trpa1*-C1 (Cat No. 400211), Mm-*Drd1*-C2 (Cat No. 461901, and Mm-*Drd2*-C3 (Cat No. 406501). Following incubation with the probes, sections were subjected to a series of amplification steps (Amp 1 at 40 °C for 30 min, Amp 2 at 40 °C for 30 min, and Amp 3 at 40 °C for 15 min). The signal was developed through probe-specific horseradish peroxidase (HRP) steps (HRP step, fluorophore step, and HRP blocker step). For nuclear staining, a DAPI solution was applied to sections for 30 s at RT. Finally, sections were coverslipped using ProLong Gold antifade mounting media and stored in the dark at 4 °C until imaging on a confocal microscope (LSM980, Carl Zeiss). Images were analyzed using a QuPath software [88]. The number of *Trpa1* puncta, the number of *Drd1*- and *Drd2*-positive cells, and co-localization of *Trpa1* with *Drd1*- or *Drd2*-positive signal were analyzed in 3 to 4 images in two sections for each animal.

### Slice preparation

For electrophysiology recordings, mice received cocaine (15 mg/kg, i.p.) injection on alternate days for 6 days related to the cocaine CPP test while control animals received saline injection for 6 consecutive days. Animals were anesthetized with isoflurane and decapitated rapidly. Their brains were rapidly removed and then submerged in ice-cold, oxygenated (95% O_2_ and 5% CO_2_), low-Ca^2+^/high-Mg^2+^ dissection buffer containing 5-mM KCl, 1.23-mM NaH_2_PO_4_, 26-mM NaHCO_3_, 10-mM dextrose, 0.5-mM CaCl_2_, 10-mM MgCl_2_ and 212.7-mM sucrose. Sagittal slices of the NAc were cut at thicknesses of 300 µm with a vibratome (VT1000S; Leica Microsystems, Heppenheim, Germany). Slices containing the NAc core and shell were kept in a holding chamber in an incubator containing oxygenated (95% O_2_ and 5% CO_2_) artificial cerebrospinal fluid containing 124-mM NaCl, 5-mM KCl, 1.23-mM NaH_2_PO_4_, 2.5-mM CaCl_2_, 1.5-mM MgCl_2_, 26-mM NaHCO_3_ and 10-mM dextrose at 28–30 °C for at least 30 min.

### Whole-cell patch-clamp recordings

Slices were transferred to a recording chamber where they were continuously perfused with artificial cerebrospinal fluid gassed with 95% O_2_ and 5% CO_2_ (flow rate: 2 ml/min) at 25 °C. Whole-cell voltage-clamp recordings were completed using a Multiclamp 700A amplifier (Molecular Devices) and slices were visualized using infrared differential interference contrast video microscopy (BX51WI; Olympus, Tokyo, Japan). Signals were filtered at 2 kHz and digitized at 10 kHz with Digidata 1440A (Molecular Devices). Patch pipettes electrodes (4–6 MΩ) contained a caesium methanesulfonate-based internal solution comprising (in mM) 130 CsMeSO_4_, 0.5 EGTA, 5 TEA-Cl, 8 NaCl, 10 HEPES, 1 QX-314, 4 Mg-ATP, 0.4 Na-GTP and 10 phosphocreatine-Na_2_ (pH, 7.2 and 285 mOsM) to record AMPAR-mediated EPSCs. The rostral and caudal limbs of the anterior commissure, as well as the caudate/putamen, were useful landmarks by which to identify the NAc core and shell neurons. MSNs of the NAc core and shell were characterized by their morphology and high resting membrane potential (−70 to −80 mV). Voltage-clamp recording was used for the characterization of synaptic transmission. sEPSCs were recorded by holding the neurons for a minimum of 2 min at −70 mV. Subsequently, sEPSC amplitude and frequency were analyzed using template analysis (Clampfit 10.7.0.3). Picrotoxin (100 μM) was always present in extracellular recording solution to block GABA_A_ receptor mediated inhibitory synaptic currents in experiments of sEPSC recording. Currents with peak amplitude smaller than 8 pA (depending on the basal noise level of the recording) were excluded from the analysis. Changes of access resistance in neurons at >20% were also excluded. Data were acquired and analyzed using pClamp 10.7.0.3 (Molecular Devices).

### Statistics

All experimental data are presented as means ± standard error of the mean. For determination of normality of samples, Shapiro-Wilk normality test was used. For determination of differences between two groups, two-tailed unpaired Student’s *t*-test was used. Data from TRPA1 KD CPP, locomotor activity, FSCV and immunoblot were analyzed by two-way ANOVA with genotype or pre-treatment as a between-subject factor and drug as a within-subject factor with Tukey’s HSD *post hoc* test. Data from TRPA1 antagonist CPP, optogenetic CPP, cocaine CPP and dopamine reuptake were analyzed by one-way ANOVA with *post hoc* Tukey’s HSD or Fisher’s LSD test. Data from behavioral sensitization and self-administration were analyzed by two-way repeated-measures ANOVA with drug treatment as a between-subject factor and time as a within-subject factor with Fisher’s LSD *post hoc* test. Data from electrophysiological experiments were analyzed by two-way ANOVA and *post hoc* Tukey’s HSD or LSD tests. Data from sEPSC, recordings in NAc MSNs between control and TRPA1 KD mice with saline or cocaine injection were analyzed by two-way ANOVA with genotype or drug treatment as well as by Tukey’s HSD. Data from the same recordings in D1R-MSNs and non-D1R-MSNs from TRPA1 KD mice with saline or cocaine injections were analyzed by two-way ANOVA with cell-type or drug treatment as a between-subject factor as well as by LSD or Tukey’s HSD *post hoc* tests. No statistical methods were used to estimate the sample size. We did not use a method of randomization to determine samples/animals allocated to experimental groups and processed.

## Supplementary information

statistics table

Supplementary information

## Data Availability

All data generated or analyzed during this study are included in this published article (and its supplementary information files), are available from the corresponding authors upon reasonable request.
