# Supplementary material for: The ion channel TRPA1 is a modulator of the cocaine reward circuit in the nucleus accumbens
Source: Mol Psychiatry. 2024 May 31;29(11):3607–22. doi: 10.1038/s41380-024-02623-4 (PMC11541219; doi:10.1038/s41380-024-02623-4)
Supplement: Supplementary file 2 — Supplementary information [file 41380_2024_2623_MOESM2_ESM.docx]

Supplementary Information for

**The ion channel TRPA1 is a modulator of the cocaine reward circuit in the nucleus accumbens**

Young-Jung Kim^1,^*, Su Jeong Choi^2,^*, Sa-Ik Hong^1,^*, Jung-Cheol Park^2,^*, Youyoung Lee^1^, Shi-Xun Ma^1^, Kwang-Hyun Hur^1^, Young Lee^2^, Kyeong-Man Kim^3^, Hyung Kyu Kim^4^, Hee Young Kim^4^, Seok-Yong Lee^1^, Se-Young Choi^2,#^, Choon-Gon Jang^1,#^

^1^Department of Pharmacology, School of Pharmacy, Sungkyunkwan University, Suwon, 16419, Republic of Korea

^2^Department of Physiology, Dental Research Institute, Seoul National University School of Dentistry, Seoul, 03080, Republic of Korea

^3^Pharmacology Laboratory, College of Pharmacy, Chonnam National University, Gwangju, 61186, Republic of Korea

^4^Department of Physiology, Yonsei University College of Medicine, Seoul, 03722, Republic of Korea

This PDF file includes:

Figure S1

Figure S2

Figure S3

Figure S4

**Supplementary Figures and Legends**


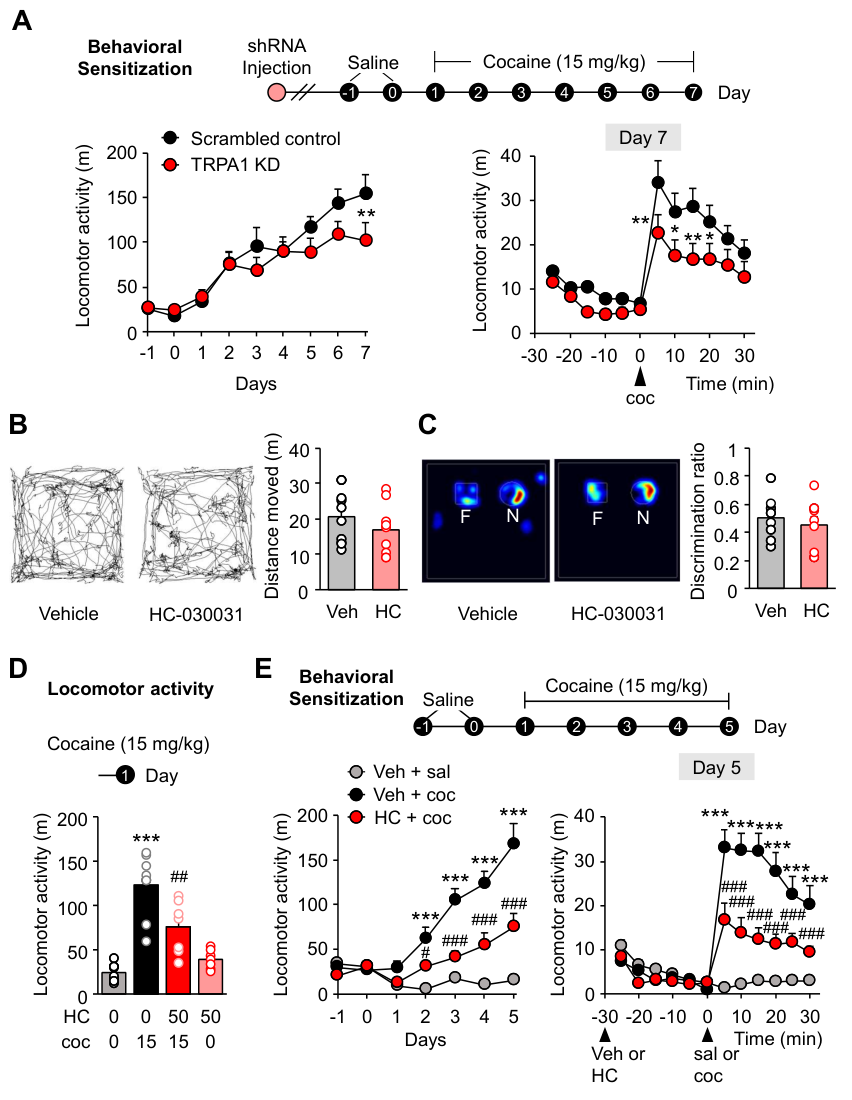


**Figure S1. Effects of TRPA1 KD and blockade on cocaine-mediated locomotor sensitization.**

**Figure S1. Effects of TRPA1 KD and blockade on cocaine-mediated locomotor sensitization.**

(A) Timeline of TRPA1 KD cocaine behavioral sensitization tests. Locomotor activity of scrambled control and TRPA1 KD groups in the cocaine behavioral sensitization tests; 30 min of total locomotor activity during 9 daily sessions [n = 8 (scrambled) and 9 (KD)].

(B) Representative trace images of locomotor activity during TRPA1 antagonist (HC-030031) open field tests. Locomotor activity on open field tests in the vehicle- or HC-030031 (10 mg/kg)-injected mice [n = 10 (vehicle) and 9 (HC-030031)].

(C) Representative heat map images of mouse movements during TRPA1 antagonist novel object recognition tests. Discrimination ratio between familiar (F) and novel (N) objects in the vehicle- or HC-030031 (10 mg/kg)-treated mice [n = 10 (vehicle) and 9 (HC-030031)].

(D) Timeline of TRPA1 antagonist acute cocaine locomotor test. 30 min of total locomotor activity of acute cocaine responses following injection of HC-030031 or vehicle [n = 8 (control), 8 (cocaine), 7 (HC-030031, cocaine), and 7 (HC-030031)].

(E) Timeline of TRPA1 antagonist cocaine behavioral sensitization tests. Locomotor activity of each group in the HC-030031 cocaine behavioral sensitization tests; 30 min of total locomotor activity during 7 daily sessions [n = 8 (control), 9 (cocaine), and 9 (HC-030031, cocaine)]. Five-min intervals of locomotor activity on day 5 [n = 8 (control), 9 (cocaine), and 9 (HC-030031, cocaine)].

Data are presented as mean ± standard error of the mean. * *P* < 0.05, ** *P* < 0.01, and *** *P* < 0.001 versus control; # *P* < 0.05, ## *P* < 0.01, and ### *P* < 0.001 versus cocaine; sal, saline; coc, cocaine; TRPA1 KD, TRPA1 knockdown; Veh, vehicle; HC, HC-030031.


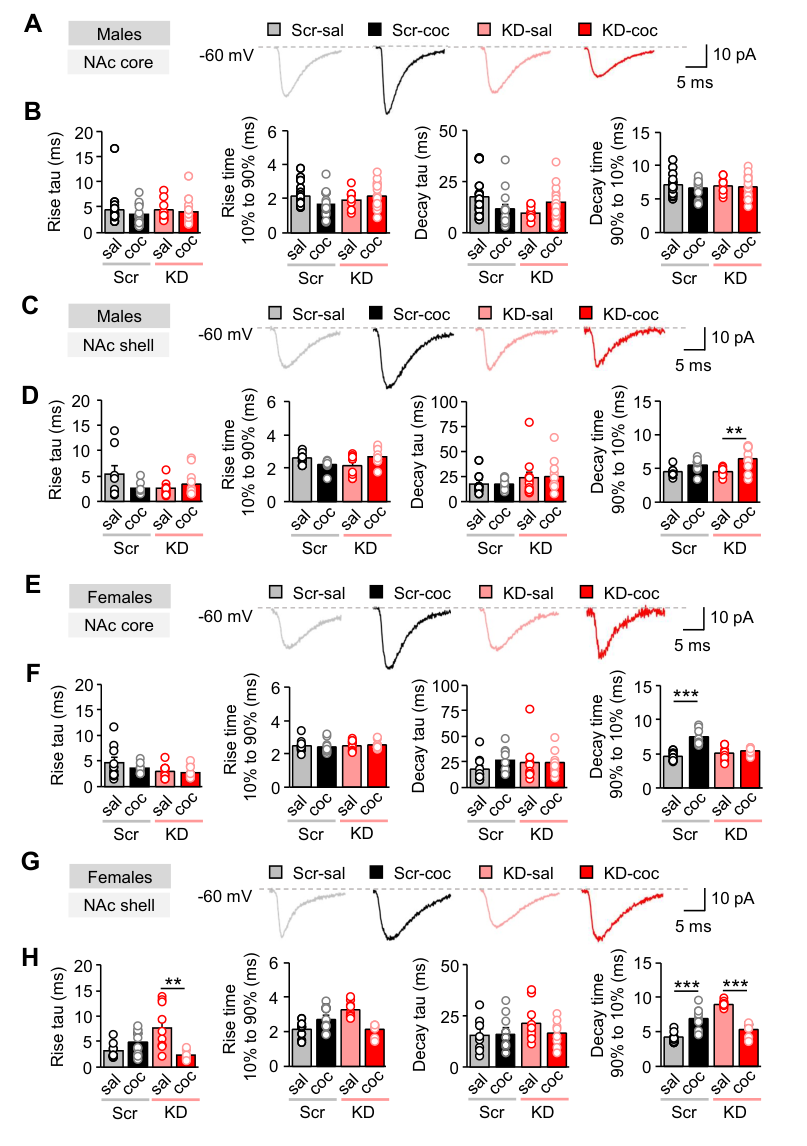


**Figure S2. Spontaneous EPSC kinetics**

**Figure S2. Spontaneous EPSC kinetics**

(A) Representative raw traces of spontaneous excitatory post-synaptic current (sEPSC) recordings from the NAc core of male mice.

(B) Rise tau (ms), rise time 10%-90% (ms), Decay tau (ms), and Decay time 90%-10% of sEPSCs in the NAc core MSNs of saline- and cocaine-injected male scrambled control and male TRPA1 KD groups [n = 16 (scrambled, saline), 13 (scrambled, cocaine), 9 (KD, saline), and 15 (KD, cocaine)].

(C) Representative raw traces of spontaneous excitatory post-synaptic current (sEPSC) recordings from the NAc shell of male mice.

(D) Rise tau (ms), rise time 10%-90% (ms), Decay tau (ms), and Decay time 90%-10% of sEPSCs in the NAc shell MSNs of saline- and cocaine-injected male scrambled control and male TRPA1 KD groups [n = 8 (scrambled, saline), 10 (scrambled, cocaine), 11 (KD, saline), and 11 (KD, cocaine)].

(E) Representative raw traces of spontaneous excitatory post-synaptic current (sEPSC) recordings from the NAc core of female mice.

(F) Rise tau (ms), rise time 10%-90% (ms), Decay tau (ms), and Decay time 90%-10% of sEPSCs in the NAc core MSNs of saline- and cocaine-injected female scrambled control and female TRPA1 KD groups [n = 10 (scrambled, saline), 9 (scrambled, cocaine), 9 (KD, saline), and 9 (KD, cocaine)].

(G) Representative raw traces of spontaneous excitatory post-synaptic current (sEPSC) recordings from the NAc shell of female mice.

(H) Rise tau (ms), rise time 10%-90% (ms), Decay tau (ms), and Decay time 90%-10% of sEPSCs in the NAc shell MSNs of saline- and cocaine-injected female scrambled control and female TRPA1 KD groups [n = 8 (scrambled, saline), 10 (scrambled, cocaine), 10 (KD, saline), and 8 (KD, cocaine)].

Data are presented as mean ± standard error of the mean. ** *P* < 0.01 and *** *P* < 0.001; sal, Saline; coc, cocaine; Scr, Scrambled control; KD, TRPA1 knockdown.


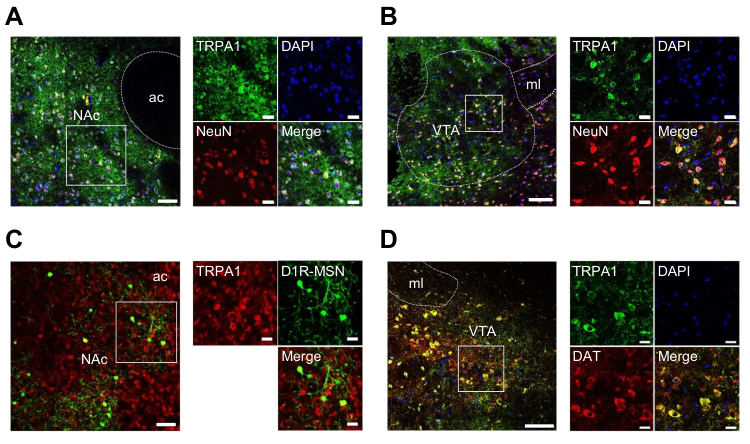


**Figure S3. Localization of TRPA1 expression in the NAc and VTA neurons**.

(A) Left: NAc neurons co-immunolabeled for TRPA1 (green), NeuN (red), and DAPI (blue). Scale bar: 50 μm. ac, anterior commissure. Right: high magnification of the NAc section. Scale bars: 20 μm.

(B) Left: VTA neurons co-immunolabeled for TRPA1 (green), NeuN (red), and DAPI (blue). Scale bar: 100 μm. ml, medial lemniscus. Right: high magnification of the VTA section. Scale bars: 20 μm.

(C) Left: EYFP-expressing D1R-MSNs (green) immunolabeled for TRPA1 (red) in the NAc of D1-Cre mice. Scale bar: 50 μm. Right: high magnification of the NAc section. Scale bars: 20 μm.

(D) Left: VTA dopaminergic neurons co-immunolabeled for TRPA1 (green), DAT (red), and DAPI (blue). Scale bar: 100 μm. ml, medial lemniscus. Right: high magnification of the VTA section. Scale bars: 20 μm.


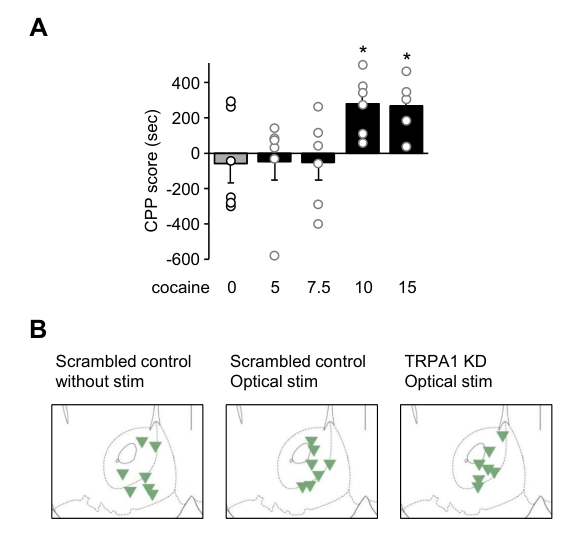


**Figure S4. Dose-response analysis of cocaine CPP histological representation for optogenetic test.**

(A) CPP score of each group in the varying doses of cocaine (5, 7.5, 10, 15 mg/kg) CPP tests [n = 6 (saline), 6 (5 mg/kg), 6 (7.5 mg/kg), 6 (10 mg/kg), and 5 (15 mg/kg)].

(B) Histology panels of AAV viral infusion regions in the NAc of each group in the TRPA1 KD optogenetic cocaine CPP tests (n = 7).

Data are presented as mean ± standard error of the mean. * *P* < 0.05 versus control; stim, stimulation; TRPA1 KD, TRPA1 knockdown.
